# Childhood trauma, antipsychotic medication, and symptom remission in first-episode psychosis

**DOI:** 10.1017/S003329172100427X

**Published:** 2023-04

**Authors:** Akiah Ottesen, W. T. V. Hegelstad, Inge Joa, Stein E. Opjordsmoen, Bjørn Rishovd Rund, Jan Ivar Røssberg, Erik Simonsen, Jan Olav Johannessen, Tor K. Larsen, Ulrik Helt Haahr, Thomas H. McGlashan, Svein Friis, Ingrid Melle

**Affiliations:** 1 NORMENT Centre, Division of Mental Health and Addiction Oslo University Hospital, Oslo, Norway; 2 Institute of Clinical Medicine, University of Oslo, Oslo, Norway; 3 Norwegian Centre for Violence and Traumatic Stress Studies, Oslo, Norway; 4 TIPS Centre for Clinical Research in Psychosis, Stavanger University Hospital, Stavanger, Norway; 5 Department of Social Studies, Faculty of Social Sciences, University of Stavanger, Stavanger, Norway; 6 Faculty of Health, Network for Medical Sciences, University of Stavanger, 4036 Stavanger, Norway; 7 Division of Mental Health and Addiction, Oslo University Hospital, Oslo, Norway; 8 Department of Psychology, University of Oslo, Oslo, Norway; 9 Vestre Viken Hospital Trust, Drammen, Norway; 10 Psychiatric Research Unit, Psychiatry Region Zealand, Slagelse, Denmark; 11 Department of Clinical Medicine, Faculty of Health and Medical Sciences, University of Copenhagen, Copenhagen, Denmark; 12 Institute of Psychiatry, University of Bergen, Bergen, Norway; 13 Department of Psychiatry, Yale University, New Haven, CT, USA

**Keywords:** Anti-psychotic medication, childhood interpersonal trauma, first-episode psychosis, outcome, symptomatic remission

## Abstract

**Background:**

To what extent psychotic symptoms in first-episode psychosis (FEP) with a history of childhood interpersonal trauma (CIT) are less responsive to antipsychotic medication is not known. In this longitudinal study, we compare symptom trajectories and remission over the first 2 years of treatment in FEP with and without CIT and examine if differences are linked to the use of antipsychotics.

**Methods:**

FEP (*N* = 191) were recruited from in- and outpatient services 1997–2000, and assessed at baseline, 3 months, 1 and 2 years. Inclusion criteria were 15–65 years, actively psychotic with a DSM-IV diagnosis of psychotic disorder and no previous adequate treatment for psychosis. Antipsychotic medication is reported as defined daily dosage (DDD). CIT (<18) was assessed with the Brief Betrayal Trauma Survey, and symptomatic remission based on scores from the Positive and Negative Syndrome Scale.

**Results:**

CIT (*n* = 63, 33%) was not associated with symptomatic remission at 2 years follow-up (71% in remission, 14% in relapse), or time to first remission (CIT 12/ no-CIT 9 weeks, *p* = 0.51). Those with CIT had significantly more severe positive, depressive, and excited symptoms. FEP with physical (*N* = 39, 20%) or emotional abuse (*N* = 22, 14, 7%) had higher DDD at 1 year (*p* < 0.05). Mean DDD did not excerpt a significant between-group effect on symptom trajectories of positive symptoms.

**Conclusion:**

Results indicate that antipsychotic medication is equally beneficial in the achievement of symptomatic remission in FEP after 2 years independent of CIT. Still, FEP patients with CIT had more severe positive, depressive, and excited symptoms throughout.

## Introduction

Epidemiological studies have confirmed an association between childhood trauma and psychotic psychopathology, including both psychotic-like experiences in the general population and increased psychotic symptoms in people with psychotic disorders (van Nierop et al., 2015; Varese et al., 2012). Childhood interpersonal trauma (CIT) is specifically potent in eliciting psychotic psychopathology (Misiak et al., 2017; van Nierop et al., 2014) more specifically hallucinations and delusions (Bailey et al., 2018; Hamner et al., 2000; Longden, Madill, & Waterman, 2012; Sun et al., 2018; Varese, Barkus, & Bentall, 2012; Vila-Badia et al., 2021). CITs are also associated with an admixture of other types of symptoms, including affective- and anxiety symptoms both in people with psychotic and non-psychotic disorders (van Nierop et al., 2016). A recent meta-analysis found that childhood trauma is correlated with the severity of both hallucinations and delusions, and further differentiation reveals that the severity of childhood neglect is associated with negative symptoms (Bailey et al., 2018). This could suggest that there may be a specificity between different forms of CIT and symptoms of psychosis. First-episode psychosis (FEP) patients exposed to CIT have been reported to have more severe positive symptoms during the first 2 years of treatment (Pruessner et al., 2021) than unexposed. Studies also suggest slower improvement and higher persistence of psychotic experiences (Trotta, Murray, & Fisher, 2015) and depressive symptoms (Aas et al., 2016) in FEP with CIT. Following, specific CIT may be associated with specific symptoms as well as outcome (Longden, Sampson, & Read, 2016), but the outcome is associated with many factors, including among others, treatment with antipsychotic medication.

To what extent psychotic symptoms in FEP with CIT are less responsive to antipsychotic medication is not known. One study found that first-episode schizophrenia patients categorized as non-responders to antipsychotic treatment more often had a history of CIT, especially emotional abuse, than responders (Misiak & Frydecka, 2016). Another study found no significant difference in reports of childhood trauma between responders and non-responders with FEP, but that both groups had more childhood trauma than healthy controls (Mondelli et al., 2015). This study also found a higher concentration of inflammatory markers in those with childhood trauma which was predictive of non-responses to anti-psychotics. A third study found that non-compliance after 1 year of treatment in FEP, a possible secondary basis for treatment non-response, was associated with an experience of parental separation in childhood (Trotta et al., 2016). Finally, treatment resistance is also found to be associated with stressful life events in general, but not CIT *per se*, after correcting for demographic characteristics associated with treatment non-response (Hassan & De Luca, 2015). We have not been able to identify any studies that have examined the association between characteristics of antipsychotic medication use and symptom development including symptom remission in FEP with, and without, CIT.

In the current naturalistic study, we aim to investigate the development of psychotic- and other symptoms, as well as the proportion achieving symptom remission in the first 2 years of treatment in FEP with, and without, CIT. We further aim to examine if differences in symptom trajectories and in rates of symptom remission are based on different antipsychotic medication use between these two groups, considering possible specificity between the type of CIT and symptom expression.

## Method

The study sample consists of FEP patients recruited from in-and outpatient services in four different Scandinavian healthcare sectors (North and South Rogaland, Oslo, Norway and Roskilde, Denmark) (Melle et al., 2004). FEP definition was not having a previous history of psychosis as ascertained by all available information i.e. interview and patient files, and not having previously received adequate treatment for psychosis. Adequate treatment is defined as antipsychotic medication of >3.5 haloperidol equivalents for >12 weeks or until remission of the psychotic symptoms. Some patients have received antipsychotics previously, but in too low doses or for a short period to have any effect on their psychotic episode – i.e. they have not received adequate treatment. This definition is a crossover between two categories often applied within this area of research (Breitborde, Srihari, & Woods, 2009).

Other inclusion criteria were age 18 (15 in Rogaland) to 65 years; meeting the DSM-IV criteria for schizophrenia, schizophreniform disorder, schizoaffective disorder, affective psychosis with mood-incongruent psychotic symptoms or other psychotic disorders (brief psychotic episode, delusional disorder, or psychotic disorder not otherwise specified); being actively psychotic as measured by the Positive and Negative Syndrome Scale (PANSS) score of 4 or more on at least one of the items 1 (delusions), 3 (hallucinatory behavior), 5 (grandiosity), or 6 (suspiciousness/persecution) or general subscale item 9 (unusual thought content); having no neurological or endocrine disorders that could affect the CNS; speaking a Scandinavian language; having an IQ score of above 70, and being willing to give informed consent. Participants with organic- or substance-induced psychosis were not included. Participants received a broad clinical assessment battery at baseline, 1 and 2 years; and an assessment of clinical symptoms at 3 months follow-up and at first remission or at relapse. Participants were treated according to a standard treatment protocol for the first 2 years which included antipsychotic medication, supportive psychotherapy and multifamily psychoeducation. For more details of the study methodology see Hegelstad et al. (2012) and Johannessen et al. (2001). The TIPS study is approved by the Regional Committee for Medical Research Ethics and the Norwegian Data Inspectorate, and the research methodology conformed to The Code of Ethics of the World Medical Association, the Helsinki Declaration of 1975 revised in 2008.

A total of 301 FEP patients were included in the TIPS study at baseline, 240 of which were assessed at 2 years follow up. Assessment of CIT was included at 5-year follow up in which 198 persons from all inclusion centers participated. The current sample is 191 participants who had full data sets including reports of CIT. There were no significant differences between the 301 and the 191 in gender distribution, age, and number with a baseline diagnosis of schizophrenia, PANSS symptoms, duration of untreated psychosis (DUP) (non-parametric) or the use of antipsychotic medication. There were significantly fewer participants with drug abuse (*N* = 8, 4.3%) in our sample than in the group that discontinued participation at 5-year inclusion (*N* = 10, 14.1%; X^2^ *=* 10.730, df 3, *p* 0.013, Standardized residual = 2.2), but no significant differences in alcohol abuse or addiction or drug addiction.

### Childhood trauma

Childhood trauma was assessed through the interview version of the Brief Betrayal Trauma Survey (BBTS) which is a 12-item, self-report measure (Goldberg & Freyd, 2006). This instrument assesses traumatic events experienced in both childhoods (<18 y), and adulthood (>18 y) in four categories of traumatic experiences: non-interpersonal trauma, interpersonal trauma by someone close or -not close to the person and other types of trauma. Test–retest stability after 3 years of childhood experiences is considerable in both women and men (Goldberg & Freyd, 2006). CIT is defined as experiencing before the age of 18 physical abuse by someone close or non-close (PA), sexual abuse by someone close or non-close (SA) or emotional abuse by someone close (EA) (Haahr et al., 2018).

### Medication

The patients who entered the study were treated with a pre-defined algorithm for antipsychotic medication for 2 years (for a detailed description see Opjordsmoen et al., 2009). Of the 301 participants at baseline, 39.2% received a first-generation antipsychotic, 57.8% a second-generation antipsychotic and 3% received no antipsychotic during the first 2 years of treatment. Here we report on the prescription of anti-psychotics using defined daily dose (DDD) of the main antipsychotic medication at baseline, 3 months, 1- and 2 year, in addition to the mean DDD over 2 years. DDD is the assumed average maintenance dose per day for a drug used for its main indication in adults (WHO Collaborating Centre for Drug Statistics Methodology, 2013). We also report any terminations of antipsychotic use, the number of changes in the type of antipsychotics used as well as the length of antipsychotic treatment in weeks. The prescription and use of other psychotropic medication was based solely on clinical judgment. Use of and type of medication was registered but not type of substance, dosage or length of treatment.

### Clinical assessment

The diagnosis was evaluated using The Structured Clinical Interview for DSM-IV Axis I Disorders SCID-I (First, Gibbon, & Williams, 1995) administered by a specifically trained clinical psychologist or medical doctors/psychiatrists. To assess current symptoms at all-time points, including at remission or relapse, participants were interviewed with the PANSS. Here we present the results of PANSS using Wallwork's five-factor model (Wallwork, Fortgang, Hashimoto, Weinberger, & Dickinson, 2012). Symptom remission was defined using the Remission criteria of the Schizophrenia Working Group Consensus (Andreasen et al., 2005); i.e. a severity score of mild or less (⩽ 3) on the following eight PANSS items: delusions, conceptual disorganization, hallucinatory behavior, blunted effect, passive/apathetic withdrawal, lack of spontaneity and flow in conversation, mannerism and posturing, and unusual thought content. DUP was defined as the time from the first week with psychotic symptoms until the start of adequate treatment. All raters were trained to reliability in the use of study instruments by rating case notes and audiotapes/videotapes, including ratings of actual videotaped interviews with participants. Reliability was considered fair to very good [DUP 0.99; PANSS positive sum-score 0.88; PANSS negative sum-score 0.76; PANSS general sum score 0.56 (ICC 1.1); kappa was 0.76 for diagnostic categories].

### Statistical analyses

We used SPSS version 26 (IBM, 2019) for statistical analyses. For bivariate between-group comparisons, we applied *t* tests for continuous variables, Pearsons' chi-square for categorical variables and non-parametric tests for DUP which violates the assumption of normality. For chi-square with more than two-way analyses, we used standardized residuals as posthoc tests with those above 2.00 interpreted as a statistically significant difference at the 0.05 level from the expected count for that category. When participants were not using any antipsychotic medication at a given time point, we entered the value = 0 instead of missing before we calculated mean DDD over the first 2 years to be used as a covariate in the ANCOVAs.

To answer our main research questions, we applied two-way ANOVA for repeated measures, with a change of symptoms over time as the within-subject variable and CIT as the between-group variable. Missing values were deleted listwise. When the effect of CIT was statistically significant, we proceeded with follow-up analysis for the subgroups of physical abuse, sexual abuse or emotional neglect as the between-subject variables. Assumptions were met in that the sample was randomly selected from the population and was large enough (*N* > 30) to analyze scales that were not normally distributed such as the PANSS. We report on the multivariate test of Greenhouse Geisser which applies a more valid critical *F*-value in cases where there is a violation of sphericity (variances are not equal between groups). We report significant variance in between-subject scores, with the null hypothesis that CIT does not have any effects on the change in symptoms over time in FEP. In cases where the null hypothesis was rejected for CIT or subgroups we then controlled for the effect of antipsychotic medication (the mean DDD from baseline to 2 years) by applying an analysis of covariance (ANCOVA). Further, we controlled for age, gender, DUP and a diagnosis within the schizophrenia spectrum disorders (schizophrenia, schizophreniform, schizoaffective disorder) at year 2. DUP was log-transformed for these analyses. Differences in time to remission was calculated using Kaplan−Meyer survival analysis with Mantel−Cox log-rank statistics.

## Results

There were 191 participants in this study (see Table 1 for characteristics). Sixty percent (*N* = 114) were male, and the mean age at baseline was 28 years (s.d. 9.5). Average years of education was 12.0 with a standard deviation of 2.4 (*N* = 183). At baseline 121 (63%) received a narrow-schizophrenia spectrum diagnosis (schizophrenia, schizophreniform or schizoaffective disorder), increasing to 140 (73%) at 2 years. Diagnostic distribution after 2 years was schizophrenia (*N* = 105, 55%), schizophreniform disorder (*N* = 8, 4.2%), schizoaffective disorder (*N* = 27, 14.1%), incongruent affective psychosis (*N* = 27, 14.1%), and other psychosis including paranoid, brief and psychosis NOS (*N* = 24, 12.6%). Median DUP was 8 weeks (range 0–1196). Seven percent had an alcohol abuse (*N* = 13) and 1% alcohol addiction (*N* = 2), while 4.3% (*N* = 8) had any drug abuse and 5.4% (*N* = 10) any drug addiction at 2 years.

**Table 1.** Sociodemographic and clinical characteristics

	Total (*n* = 191)	CIT (*n* = 63)	No-CIT (*n* = 128)	*T*-test	
Age	27.9 ± 9.5	27.9 ± 8.9	27.9 ± 9.9	−0.050	ns
Education (*N* = 183)	12.1 ± 2.4	11.8 ± 2.3	12.2 ± 2.5	1.094	ns
				Chi-square	
Male	114 (59.7%)	36 (31.6%)	78 (60.9%)	0.615	ns
Schizophrenia spectrum	121 (63.4%)	42 (66.7%)	79 (61.7%)	0.505	ns
				Mann–Whitney U	
DUP	Mdn 8 IQR 1196	Mdn 8 IQR 1196	Mdn 7 IQR 520	4238.0	ns

### Childhood interpersonal trauma

Of the 191 participants, 33% (*N* = 63) reported any CIT; 20% (*N* = 39) PA, 11.5% (*N* = 22) SA and 14.7% (*N* = 28) EA. As can be seen in Table 1 there were no significant differences in age, education level, gender, diagnosis or DUP between those reporting CIT and those not. There was a statistically significant correlation between the presence of PA and EA (*r* = 0.450, *p* < 0.001) with 29% of those with CIT reporting both experiences. There was no statistically significant correlation between SA with either PA or EA. Significantly more females (*N* = 17, 22%) reported SA than males (*N* = 5, 4%) (X^2^ = 14.403, df1, *p* < 0.001), while slightly more males reported EA (*N* = 21, 18%) than females (*N* = 7, 9%) however without reaching the level of statistical significance (x^2^ = 3079, df 1, *p* = 0.08). Those who reported EA had significantly less education than those without EA, with a mean of 10.8 years (s.d. 2.2) compared to 12.3 (s.d. 2.4) (*t*-test = 2950, df 180, *p* < 0.004). We found no significant differences between participants reporting PA, SA or EA for age, DUP or diagnosis at baseline and at 2-year follow-up.

### Use of medications

Less than 2% of the participants (*N* = 3) were not using antipsychotics at baseline. Olanzapine was the most used antipsychotic (*N* = 89, 47% of the cases), followed by perphenazine (*N* = 48, 25%), risperidone (*N* = 15, 8%) and zuclopenthixol (*N* = 14, 7%). The remaining 11 participants used a range of other medications. After 2 years, 46% (*N* = 88) had used only one (i.e. the same) medication from baseline to follow-up; 43% (*N* = 89) had changed medication once, 10% (*N* = 19) twice and 1% (*N* = 2) three times. Nearly 30% (*N* = 56) had stopped using antipsychotic medication at 2 years follow-up. The mean length of antipsychotic medication use was 74 weeks (s.d. 31). A total of 43% used anti-depressive medication at some time during the treatment period, and a total of 13% used mood stabilizers.

### CIT and antipsychotic medication

We did not find any associations between CIT and discontinuation of antipsychotic use, changes *v.* stability in the use of antipsychotic medication, or the number of different antipsychotic medications that had been used (Table 2). Those reporting CIT used significantly lower DDDs at baseline compared to those without CIT. At 2-year follow-up, those reporting CIT had an increased dosage and no longer differed from those without CIT. Focusing on subgroups, those reporting PA and EA used significantly higher DDDs at 12 months than those without. Also, those reporting EA had a significantly longer duration of their antipsychotic medication use compared to those without EA.

**Table 2.** Comparison of anti-psychotic use in the first 2 years of treatment of FEP between those with or without CIT before the age of 18

Anti-psychotic use		Childhood interpersonal trauma (df 189)		Physical abuse (df 189)		Sexual abuse (df 188)		Emotional abuse (df 188)	
		Mean ± s.d.	*t*-test	Mean ± s.d.	*t*-test	Mean ± s.d.	*t*-test	Mean ± s.d.	*t*-test
DDD baseline	Yes	0.52 ± 0.31	2.349*	0.50 ± 0.26	1.907	0.61 ± 0.36	0.055	0.55 ± 0.29	0.893
	No	0.67 ± 0.46		0.65 ± 0.45		0.62 ± 0.43		0.63 ± 0.44	
DDD 3 months	Yes	0.77 ± 0.63	0.308	0.86 ± 0.65	−0.829	0.82 ± 0.72	−0.291	0.97 ± 0.70	−1.722
	No	0.80 ± 0.61		0.77 ± 0.60		0.78 ± 0.60		0.75 ± 0.60	
DDD 1 year	Yes	0.81 ± 0.63	−1.537	0.89 ± 0.65	−2.072*	0.88 ± 0.74	−1.358	0.95 ± 0.71	−2.200*
	No	0.66 ± 0.62		0.66 ± 0.61		0.69 ± 0.61		0.67 ± 0.61	
DDD 2 years	Yes	0.77 ± 0.75	−0.230	0.73 ± 0.62	0.215	0.75 ± 0.91	0.055	0.87 ± 0.63	−0.879
	No	0.75 ± 0.78		0.76 ± 0.80		0.76 ± 0.75		0.73 ± 0.79	
DDD mean 0–2 years	Yes	0.72 ± 0.42	0.015	0.75 ± 0.41	−0.500	0.77 ± 0.50	−0.576	0.84 ± 0.44	−1.635
	No	0.72 ± 0.42		0.71 ± 0.42		0.71 ± 0.41		0.70 ± 0.41	
Number of changes in AP	Yes	0.71 ± 0.66	−0.756	0.7 ± 0.66	−0.326	0.55 ± 0.60	0.800	0.68 ± 0.67	−0.169
	No	0.63 ± 0.72		0.65 ± 0.71		0.67 ± 0.71		0.65 ± 0.71	
Duration of medicine in weeks^a^	Yes	79.9 ± 27.5	−1.696	78.6 ± 26.2	−0.962	78.4 ± 31.3	−0.635	85.4 ± 22.3	−1.996*
	No	71.7 ± 32.5		73.2 ± 32.3		73.8 ± 21.3		72.5 ± 32.2	
		*N* (%)	X^2^ (*df1*)	*N* (%)	X^2^ (*df1*)	*N* (%)	X^2^ (*df1*)	*N* (%)	X^2^ (*df1*)
AP discontinued at 2 years	Yes	47 (25.4)	0.698	8 (20.5)	1.834	7 (31.8)	0.066	6 (21.4)	1.023
	No	40 (31.3)		48 (31.6)		49 (29.2)		50 (30.9)	
AP stable through 2 years	Yes	25 (39.7)	0.347	16 (41)	0.240	11 (50)	0.279	12 (42.9)	0.047
	No	60 (47)		69 (45.4)		74 (44)		73 (45.1)	

a df 187 for CIT and PA; df 186 for SA and EA.

*p < 0.05.

### CIT and symptom trajectories over the follow-up period

**Fig. 1.**
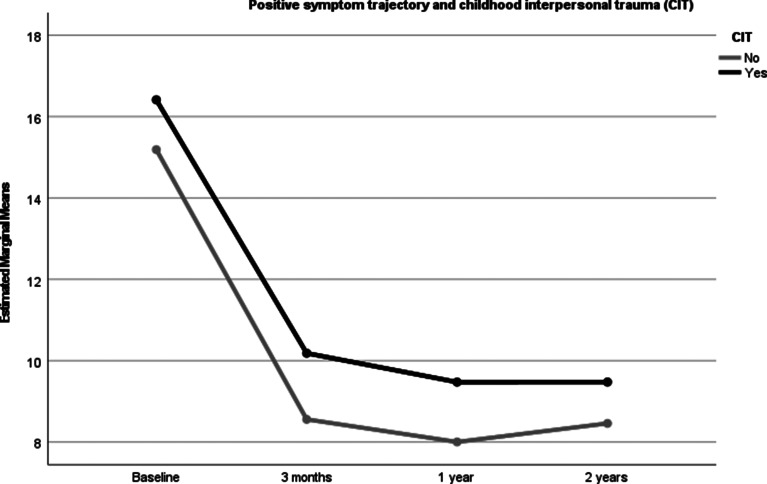
PANSS positive symptom trajectory in those with and without CIT controlling for a DDD of anti-psychotic medication.

**Fig. 2.**
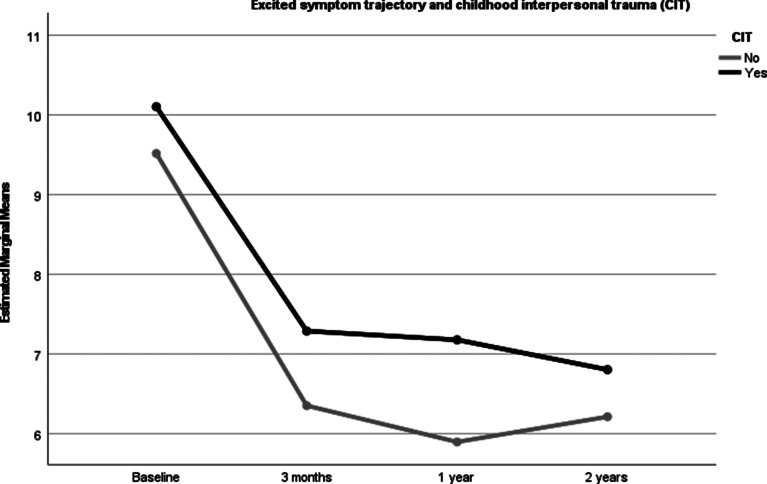
PANSS excited symptom trajectory in those with and without CIT controlling for a DDD of anti-psychotic medication.

**Fig. 3.**
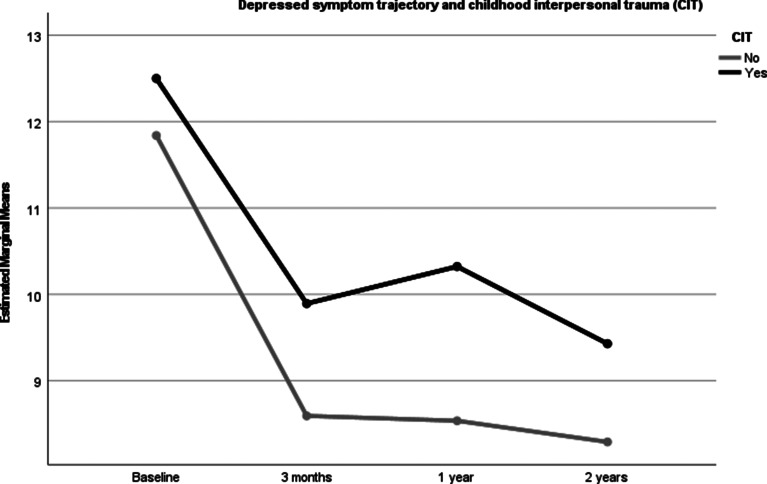
PANSS depressed symptom trajectory in those with and without CIT controlling for a DDD of anti-psychotic medication.

All symptoms decreased significantly over the follow-up period. Statistically significant CIT between-group differences were found for the level of positive, depressive and excited symptoms, with those with CIT showing more severe symptoms than those without. There were however no differences for cognitive or negative symptoms. The between-group differences remained statistically significant after controlling for the mean DDD of main antipsychotics from baseline to 2 years (Table 3, Figs 1–3). After controlling for age, gender, DUP and schizophrenia spectrum diagnosis the differences found in symptom trajectories from baseline to 2 years lost significance for excited and depressive symptoms, but still significant differences for positive symptoms over time. Between-group differences remained significant for positive, excited and depressive symptoms after controlling for all covariates.

**Table 3.** Repeated measure analysis of covariance for symptom reduction over four-time points (baseline, 3 months, 1 year and 2 years) and ANCOVA controlling for anti-psychotic dosage in DDD mean from baseline to 2 years, and gender, age, DUP and schizophrenia spectrum disorder at year 2

	ANOVA	*p*	Adj *R*^2^	ANCOVA^a^	*p*	Adj *R*^2^	ANCOVA^b^	*p*	Adj *R*^2^
**Childhood interpersonal trauma (*N* = 161)**									
Positive symptoms									
*Within*	136.879	0.001	0.463	58.509	0.001	0.270	11.510	0.001	0.070
*Between*	7.555	0.007	0.045	8.690	0.004	0.052	8.054	0.005	0.050
Excited symptoms									
*Within*	43.076	0.001	0.213	19.440	0.001	0.110	0.757	0.478	0.005
*Between*	7.985	0.005	0.048	7.942	0.005	0.048	7.829	0.006	0.048
Depressed symptoms (*N* = 162)									
*Within*	37.769	0.001	0.191	17.292	0.001	0.098	1.441	0.232	0.009
*Between*	10.164	0.002	0.060	10.435	0.002	0.062	10.346	0.002	0.063
**Physical abuse (*N* = 161)**									
Positive symptoms									
*Within*	99.601	0.001	0.385	52.298	0.001	0.249	10.999	0.001	0.067
*Between*	6.670	0.011	0.040	7.357	0.007	0.044	6.183	0.014	0.039
Excited symptoms									
*Within*	29.973	0.001	0.159	16.797	0.001	0.096	0.798	0.458	0.005
*Between*	9.108	0.003	0.054	9.127	0.003	0.055	8.003	0.005	0.049
Depressed symptoms (*N* = 162)									
*Within*	23.728	0.001	0.129	13.475	0.001	0.078	1.123	0.339	0.007
*Between*	7.016	0.009	0.042	7.070	0.009	0.043	6.912	0.009	0.043
**Emotional abuse (*N* = 160)**									
Positive symptoms									
*Within*	80.010	0.001	0.336	46.751	0.001	0.229	11.397	0.001	0.069
*Between*	3.324	0.070	0.021	1.863	0.174	0.012	1.488	0.224	0.010
Excited symptoms									
*Within*	25.648	0.001	0.140	15.657	0.001	0.091	8.202	0.709	0.502
*Between*	4.359	0.038	0.027	4.895	0.028	0.030	3.673	0.057	0.023
Depressed symptoms (*N* = 162)									
*Within*	15.190	0.001	0.087	9.982	0.001	0.059	0.803	0.485	0.005
*Between*	14.611	0.001	0.084	12.900	0.001	0.075	11.569	0.001	0.070

a Controlling for DDD mean 0–2 years.

b Controlling for DDD mean, Gender, age, DUP and Schizophrenia spectrum disorder at year 2.

Within DF = 3, Between DF = 1.

Follow up analyses reveals similar patterns for those reporting physical abuse, with significant changes in positive symptoms over the first 2 years between those with and without PA. However, this was not found for the group reporting emotional abuse who only showed higher symptoms of depression after controlling for covariates.

### CIT and symptom remission

At 2 years 69% (*N* = 132) were in remission, 10% (*N* = 19) in relapse and 21% (*N* = 40) continuously psychotic. The presence of CIT did not influence the chances of being in remission at either 1 or 2 years. There were no significant differences in chances of remission between those with and without PA, EA or SA. There were also no significant differences in time to first remission for those with- and those without CIT (12 weeks 95% CI 7.9–16.1 *v.* 9 weeks 95% CI 6.6–11.4, *p* = 0.51, Mantel−Cox log-rank statistics).

## Discussion

The main finding of this study was that experiencing CIT did not influence the chances of symptom remission at 2 years follow-up in a sample of persons with FEP. Also, the presence of CIT did not have a statistically significant influence on the time to the first remission. Both groups experienced statistically significant improvements in key symptoms with no differences in symptom trajectories. There were however differences in symptom levels as those reporting CIT had more severe positive, depressed, and excited symptoms at all-time points. Those with CIT received lower doses of antipsychotics at baseline, while those with PA and EA higher doses at 1 year and those with EA also had a longer duration of medication. Controlling for mean antipsychotic dosage did not excerpt a significant effect on between-group differences in symptom levels, suggesting that differences in symptom levels were not associated with different antipsychotic use or dosage between persons with and without CIT.

Younger age, being single and male, deteriorating premorbid social functioning, narrow schizophrenia and longer DUP predicted non-remission at 2 years in the TIPS study (Simonsen et al., 2010), and it seems that inclusion of CIT does not contribute significantly to a prognostic model of remission of psychotic symptoms. Previous research has not found a robust association between childhood trauma and illness course or symptom remission during the first year of treatment (Trotta et al., 2016), and our findings are in line with this. Another recently published study found that FEP patients with childhood trauma had lower chances of remission of positive symptoms at 1 year, but not 2 years (Pruessner et al., 2021). Even though our study did not find that CIT was associated with remission rates at either 1 or 2 years, we did find that those with childhood trauma had higher levels of positive symptoms at 2 years. The severity of these positive symptoms did not influence remission rates directly, but our findings are in line with other studies showing that childhood adversity is associated with the persistence of psychotic experiences as well as clinically relevant psychotic symptoms (Trotta et al., 2015). In the follow-up analyses, we found an association between PA and higher levels of positive symptoms, in addition to findings of a higher DDD at 1 year. More severe positive symptoms in those reporting PA have been reported in several previous studies (Bentall, Wickham, Shevlin, & Varese, 2012; Fisher et al., 2010; Murphy, Shevlin, Houston, & Adamson, 2014). Those with EA had significantly more severe depression and excited symptoms but not positive psychosis symptoms. Still, they also received a higher dosage at 1 year and were medicated for longer. The association between EA and depression in patients with psychosis is previously shown to be mediated by meta-cognitive beliefs of thoughts being uncontrollable or dangerous (Østefjells et al., 2017), suggesting that there are important psychological mechanisms that also need to be studied further. There are otherwise few reports of the association between EA and specific symptoms (see e.t. Read, van Os, Morrison, & Ross, 2005). Taken together, our findings can indicate that antipsychotic medication is beneficial in reducing symptoms in both those with and without CIT. Despite no differences in remission rates between those with and without CIT those exposed to PA and EA did have higher symptoms for some factors and higher dosage of antipsychotics at 1 year. This may suggest that they require a higher dosage of antipsychotics during the first 2 years of treatment than those without CIT, but it could also be a consequence of other factors not assessed here, such as the need for more psychoeducation, or psychological therapies in those who have experienced CIT in childhood.

We did not find any differences in the stability, discontinuation or change of antipsychotics between those with and without CIT. We did however find lower DDD at baseline for participants with CIT, but not later in the treatment course for this group as a whole, but just for those with PA and EA at 1 year. Pruessner et al. (2021) find higher antipsychotic dosage at 12 and 24 months in FEP with childhood trauma, compared to those without childhood trauma, but did not differentiate antipsychotic dosage in the different trauma subgroups. Two uncontrolled studies assessing outcome after the first 12 weeks of treatment show conflicting findings; one reports that FEP schizophrenia patients with childhood trauma were more often non-responders to medication (Misiak & Frydecka, 2016), while the other found no differences in treatment response to antipsychotics for participants with and without childhood trauma (Mondelli et al., 2015). A recent study from South Africa applying long-acting injectable antipsychotic treatment to FEP schizophrenia patients, found slower treatment response the first 2 years for positive and disorganized symptoms in those with CIT (Kilian et al., 2020). Another study of treatment outcome found that genetic variation (SNP in the MMP9 gene) was associated with the risk of developing treatment-resistant schizophrenia, with an additional interaction effect with experiences of childhood trauma (McGregor et al., 2018). As findings are inconsistent more targeted studies of the association between CIT and response to antipsychotic medications are required.

In addition to findings concerning psychotic symptoms and their remission, we also found that FEP with CIT, specifically PA and EA, had more severely depressed and excited symptoms over the first year of treatment than those without. A significant association between childhood trauma and depressed symptoms both at baseline and 1 year are previously reported in an FEP sample using the same PANSS factor model (Aas et al., 2016). Also, in a large epidemiological study of psychosis, all types of CIT were associated with lifetime depression (Turner et al., 2019). Childhood maltreatment is a predictor of depression, as well as lack of treatment response or remission of clinical depression (Nanni, Uher, & Danese, 2012). Depression can thus be perceived as an important transdiagnostic reaction to trauma, meaning it occurs across many diagnostic categories in cases where there has been childhood trauma. Internalizing symptoms such as depression are found to be the strongest concurrent and prospective predictor of disability in patients with psychotic disorder (Longenecker et al., 2021). One possible conclusion is that depressive symptoms and trauma-related reactions are an important treatment priority in people with psychosis and CIT. However, a relatively recent systematic review and meta-analysis found that trauma-focused psychological interventions did not have a significant impact on depression and anxiety in persons with psychotic disorders, and the effect on positive psychotic symptoms was small and temporary (Brand, McEnery, Rossell, Bendall, & Thomas, 2018). While CIT has a significant impact on illness burden in psychotic disorders it is still unclear how we can translate this knowledge into better medications or improved psychological interventions.

### Strengths and limitations

The primary limitations are that the study was not designed specifically to assess the effect of antipsychotic medication. The study had a medication algorithm that allowed for individual dosing, but with an upper DDD limit during the first 2 years, thus creating a potential ceiling effect. DDD's are internationally accepted measures based on a variety of sources and available for most drugs, but the limitations are that they are a standardized measure of drug consumption and not specifically a dose equivalent (Leucht, Samara, Heres, & Davis, 2016). Further, the structured medication algorithm was for antipsychotics. Patients could however receive other medications based on clinical evaluation, primarily including antidepressants and mood stabilizers in case of significant mood episodes. However, these data were considered insufficient for inclusion in the analyses.

Additionally, the validity of retrospective reports of traumatic experiences in childhood based on a possible distortion of memories as well as withholding information on trauma is discussed (Roy & Perry, 2004). A recent study found slight to fair agreement between prospective and retrospective reports of childhood trauma from different informers, but that retrospective reports from persons themselves had the highest correlation with adult mental health issues (Newbury et al., 2018). We have not included measures of bullying in adolescents in this study. As studies show that bullying is higher in those at ultra-high risk for psychosis (Peh, Rapisarda, & Lee, 2019) and that persons with FEP are twice as likely to have experiences bullying than unaffected controls (Trotta et al., 2013) this is an important experience that should be included in future studies of the association between childhood trauma, psychosis and effect of anti-psychotic medication. We did not find significant differences in standard socio-demographic and clinical variables between the study participants and those lost to follow up, but since CIT was assessed at follow-up we do not know if those lost to follow-up had more or less CIT than participants. Also, we did not control for psychotherapy or psychoeducation in this study, the effect of which may have been potent to remission in addition to antipsychotics. The repeated measure analyses used in this study only allows for the listwise deletion of data reducing our sample for these analyses. This is also a limitation to the study as missing data may not be completely at random. Finally, the study is explorative in nature and the subgroups can be considered small. This is a limitation as it increases the risk of both type I and II errors. However, as this is a new area of research we believe that our findings are an important contribution to the field concerned with the treatment of psychotic symptoms in persons with FEP and CIT.

Study strengths include recruitment of a representative study sample early in the treated course of illness and a study protocol including structured and well-defined treatment approaches. The study was also conducted in Scandinavian countries which provide socialized medicine offering the same treatment to all citizens independent of socio-demographic background and is thus a good representation across the whole population.

## Conclusion

We found no differences in the rate of- and time to remission of psychotic symptoms between participants with and without CIT. Only a small variation in anti-psychotics was found with CIT receiving lower doses at baseline, and those with PA and EA higher doses at 1 year. CIT was associated with more severe positive, excited and depressive symptoms, and correction for differences in medications did not influence these associations. Our findings indicate that antipsychotic medication is beneficial in reducing positive symptoms in both those with and without CIT. However FEP patients with CIT still have more severe positive, depressive and excited symptoms during the first 2 years of treatment, and discovering adequate treatments for these symptoms is an important priority in the future.

## References

[ref1] Aas, M., Andreassen, O. A., Aminoff, S. R., Færden, A., Romm, K. L., Nesvåg, R., … Melle, I. (2016). A history of childhood trauma is associated with slower improvement rates: Findings from a one-year follow-up study of patients with a first-episode psychosis. BMC Psychiatry, 16, 126–126. doi: 10.1186/s12888-016-0827-4 27146044 PMC4855869

[ref2] Andreasen, N. C., Carpenter, W. T. Jr., Kane, J. M., Lasser, R. A., Marder, S. R., & Weinberger, D. R. (2005). Remission in schizophrenia: Proposed criteria and rationale for consensus. American Journal of Psychiatry, 162(3), 441–449. doi:10.1176/appi.ajp.162.3.441 15741458

[ref3] Bailey, T., Alvarez-Jimenez, M., Garcia-Sanchez, A. M., Hulbert, C., Barlow, E., & Bendall, S. (2018). Childhood trauma is associated with severity of hallucinations and delusions in psychotic disorders: A systematic review and meta-analysis. Schizophrenia Bulletin, 44(5), 1111–1122. doi: 10.1093/schbul/sbx161 29301025 PMC6101549

[ref4] Bentall, R. P., Wickham, S., Shevlin, M., & Varese, F. (2012). Do specific early-life adversities lead to specific symptoms of psychosis? A study from the 2007 the adult psychiatric morbidity survey. Schizophrenia Bulletin, 38(4), 734–740. doi: 10.1093/schbul/sbs049 22496540 PMC3406525

[ref5] Brand, R. M., McEnery, C., Rossell, S., Bendall, S., & Thomas, N. (2018). Do trauma-focussed psychological interventions have an effect on psychotic symptoms? A systematic review and meta-analysis. Schizophrenia Research, 195, 13–22. doi: 10.1016/j.schres.2017.08.037 28844432

[ref6] Breitborde, N. J., Srihari, V. H., & Woods, S. W. (2009). Review of the operational definition for first-episode psychosis. Early Intervention in Psychiatry, 3(4), 259–265. doi: 10.1111/j.1751-7893.2009.00148.x 22642728 PMC4451818

[ref7] First, M. S. R., Gibbon, M., & Williams, J. (1995). Structured clinical interview for DSM-IV axis I disorders. Patient Edition SCID I/P (Version 2.0 ed ed.). New York, NY: New York State Psychiatric Institute, Biometrics Research Department.

[ref8] Fisher, H. L., Jones, P. B., Fearon, P., Craig, T. K., Dazzan, P., Morgan, K., … Morgan, C. (2010). The varying impact of type, timing and frequency of exposure to childhood adversity on its association with adult psychotic disorder. Psychological Medicine, 40(12), 1967–1978. doi: 10.1017/S0033291710000231 20178679 PMC3272393

[ref9] Goldberg, L. R., & Freyd, J. J. (2006). Self-reports of potentially traumatic experiences in an adult community sample: Gender differences and test-retest stabilities of the items in a brief betrayal-trauma survey. Journal of Trauma & Dissociation, 7(3), 39–63. doi: 10.1300/J229v07n03_04, 16873229

[ref10] Haahr, U. H., Larsen, T. K., Simonsen, E., Rund, B. R., Joa, I., Rossberg, J. I., … Melle, I. (2018). Relation between premorbid adjustment, duration of untreated psychosis and close interpersonal trauma in first-episode psychosis. Early Intervention in Psychiatry, 12(3), 316–323. doi: 10.1111/eip.12315 26800653

[ref11] Hamner, M. B., Frueh, B. C., Ulmer, H. G., Huber, M. G., Twomey, T. J., Tyson, C., & Arana, G. W. (2000). Psychotic features in chronic posttraumatic stress disorder and schizophrenia: Comparative severity. Journal of Nervous and Mental Disorders, 188(4), 217–221. 10.1097/00005053-200004000-00004 10789998

[ref12] Hassan, A. N., & De Luca, V. (2015). The effect of lifetime adversities on resistance to antipsychotic treatment in schizophrenia patients. Schizophrenia Research, 161(2–3), 496–500. doi: 10.1016/j.schres.2014.10.048 25468176

[ref13] Hegelstad, W. T. V., Larsen, T. K., Auestad, B., Evensen, J., Haahr, U., Joa, I., … McGlashan, T. (2012). Long-term follow-up of the TIPS early detection in psychosis study: Effects on 10 year outcome. American Journal of Psychiatry, 169(4), 374–380. doi: 10.1176/appi.ajp.2011.11030459 22407080

[ref14] IBM (2019). IBM SPSS statistics for windows *(*version 26.0*)*. Armonk, NY: IBM Corp.

[ref15] Johannessen, J. O., McGlashan, T. H., Larsen, T. K., Horneland, M., Joa, I., Mardal, S., … Vaglum, P. (2001). Early detection strategies for untreated first-episode psychosis. Schizophrenia Research, 51(1), 39–46. doi: 10.1016/s0920-9964(01)00237-7 11479064

[ref16] Kilian, S., Asmal, L., Phahladira, L., Plessis, S. D., Luckhoff, H., Scheffler, F., … Emsley, R. (2020). The association between childhood trauma and treatment outcomes in schizophrenia spectrum disorders. Psychiatry Research, 289, 113004. doi: 10.1016/j.psychres.2020.113004 32387789

[ref17] Leucht, S., Samara, M., Heres, S., & Davis, J. M. (2016). Dose equivalents for antipsychotic drugs: The DDD method. Schizophrenia Bulletin, 42(Suppl 1), S90–S94. doi: 10.1093/schbul/sbv167 27460622 PMC4960429

[ref18] Longden, E., Madill, A., & Waterman, M. G. (2012). Dissociation, trauma, and the role of lived experience: Toward a new conceptualization of voice hearing. Psychological Bulletin, 138(1), 28–76. doi: 10.1037/a0025995 22082488

[ref19] Longden, E., Sampson, M., & Read, J. (2016). Childhood adversity and psychosis: Generalised or specific effects? Epidemiology and Psychiatric Sciences, 25(4), 349–359. doi: 10.1017/s204579601500044x 26156083 PMC7137611

[ref20] Longenecker, J., Bagby, R., McKenzie, K., Pollock, B., George, T., Voore, P., & Quilty, L. (2021). Cross-cutting symptom domains predict functioning in psychotic disorders. Journal of Clinical Psychiatry, 82(2), 20m13288. 10.4088/JCP.20m13288 33988932

[ref21] McGregor, N., Thompson, N., O'Connell, K. S., Emsley, R., van der Merwe, L., & Warnich, L. (2018). Modification of the association between antipsychotic treatment response and childhood adversity by MMP9 gene variants in a first-episode schizophrenia cohort. Psychiatry Research, 262, 141–148. doi: 10.1016/j.psychres.2018.01.044 29448178

[ref22] Melle, I., Larsen, T. K., Haahr, U., Friis, S., Johannessen, J. O., Opjordsmoen, S., … McGlashan, T. (2004). Reducing the duration of untreated first-episode psychosis: Effects on clinical presentation. Archives of General Psychiatry, 61(2), 143–150. doi: 10.1001/archpsyc.61.2.143 14757590

[ref23] Misiak, B., & Frydecka, D. (2016). A history of childhood trauma and response to treatment with antipsychotics in first-episode schizophrenia patients: Preliminary results. Journal of Nervous and Mental Disorder, 204(10), 787–792. doi: 10.1097/nmd.0000000000000567 27441460

[ref24] Misiak, B., Krefft, M., Bielawski, T., Moustafa, A. A., Sąsiadek, M. M., & Frydecka, D. (2017). Toward a unified theory of childhood trauma and psychosis: A comprehensive review of epidemiological, clinical, neuropsychological and biological findings. Neuroscience & Biobehavioral Reviews, 75, 393–406. doi: 10.1016/j.neubiorev.2017.02.015 28216171

[ref25] Mondelli, V., Ciufolini, S., Belvederi Murri, M., Bonaccorso, S., Di Forti, M., Giordano, A., … Dazzan, P. (2015). Cortisol and inflammatory biomarkers predict poor treatment response in first-episode psychosis. Schizophrenia Bulletin, 41(5), 1162–1170. doi: 10.1093/schbul/sbv028 25829375 PMC4535637

[ref26] Murphy, J., Shevlin, M., Houston, J., & Adamson, G. (2014). Modelling the co-occurrence of psychosis-like experiences and childhood sexual abuse. Social Psychiatry and Psychiatric Epidemiology, 49(7), 1037–1044. doi: 10.1007/s00127-014-0845-9 24562388

[ref27] Nanni, V., Uher, R., & Danese, A. (2012). Childhood maltreatment predicts unfavorable course of illness and treatment outcome in depression: A meta-analysis. American Journal of Psychiatry, 169(2), 141–151. doi: 10.1176/appi.ajp.2011.11020335 22420036

[ref28] Newbury, J. B., Arseneault, L., Moffitt, T. E., Caspi, A., Danese, A., Baldwin, J. R., & Fisher, H. L. (2018). Measuring childhood maltreatment to predict early-adult psychopathology: Comparison of prospective informant-reports and retrospective self-reports. Journal of Psychiatric Research, 96, 57–64. doi: 10.1016/j.jpsychires.2017.09.020 28965006 PMC5725307

[ref29] Opjordsmoen, S., Melle, I., Friis, S., Haahr, U., Johannessen, J. O., Larsen, T. K., … McGlashan, T. H. (2009). Stability of medication in early psychosis: A comparison between second-generation and low-dose first-generation antipsychotics. Early Intervention in Psychiatry, 3(1), 58–65. doi: 10.1111/j.1751-7893.2008.00103.x 21352176

[ref30] Østefjells, T., Lystad, J. U., Berg, A. O., Hagen, R., Loewy, R., Sandvik, L., … Røssberg, J. I. (2017). Metacognitive beliefs mediate the effect of emotional abuse on depressive and psychotic symptoms in severe mental disorders. Psychological Medicine, 47(13), 2323–2333. doi: 10.1017/S0033291717000848 28397634

[ref31] Peh, O. H., Rapisarda, A., & Lee, J. (2019). Childhood adversities in people at ultra-high risk (UHR) for psychosis: A systematic review and meta-analysis. Psychological Medicine, 49(7), 1089–1101. doi: 10.1017/S003329171800394X 30616701

[ref32] Pruessner, M., King, S., Veru, F., Schalinski, I., Vracotas, N., Abadi, S., … Joober, R. (2021). Impact of childhood trauma on positive and negative symptom remission in first-episode psychosis. Schizophrenia Research, 231, 82–89. doi: 10.1016/j.schres.2021.02.023 33812301

[ref33] Read, J., van Os, J., Morrison, A. P., & Ross, C. A. (2005). Childhood trauma, psychosis and schizophrenia: A literature review with theoretical and clinical implications. Acta Psychiatrica Scandinavica, 112(5), 330–350. doi: 10.1111/j.1600-0447.2005.00634.x 16223421

[ref34] Roy, C. A., & Perry, J. C. (2004). Instruments for the assessment of childhood trauma in adults. Journal of Nervous and Mental Disease, 192(5), 343–351. 15126888 10.1097/01.nmd.0000126701.23121.fa

[ref35] Simonsen, E., Friis, S., Opjordsmoen, S., Mortensen, E. L., Haahr, U., Melle, I., … McGlashan, T. H. (2010). Early identification of non-remission in first-episode psychosis in a two-year outcome study. Acta Psychiatrica Scandinavica, 122(5), 375–383. doi: 10.1111/j.1600-0447.2010.01598.x 20722632

[ref36] Sun, P., Alvarez-Jimenez, M., Simpson, K., Lawrence, K., Peach, N., & Bendall, S. (2018). Does dissociation mediate the relationship between childhood trauma and hallucinations, delusions in first-episode psychosis? Comprehensive Psychiatry, 84, 68–74. doi: 10.1016/j.comppsych.2018.04.004 29694935

[ref37] Trotta, A., Di Forti, M., Mondelli, V., Dazzan, P., Pariante, C., David, A., … Fisher, H. L. (2013). Prevalence of bullying victimisation amongst first-episode psychosis patients and unaffected controls. Schizophrenia Research, 150(1), 169–175. doi: 10.1016/j.schres.2013.07.001 23891482 PMC3825661

[ref38] Trotta, A., Murray, R. M., David, A. S., Kolliakou, A., O'Connor, J., Di Forti, M., … Fisher, H. L. (2016). Impact of different childhood adversities on 1-year outcomes of psychotic disorder in the genetics and psychosis study. Schizophrenia Bulletin, 42(2), 464–475. doi: 10.1093/schbul/sbv131 26373540 PMC4753600

[ref39] Trotta, A., Murray, R. M., & Fisher, H. L. (2015). The impact of childhood adversity on the persistence of psychotic symptoms: A systematic review and meta-analysis. Psychological Medicine, 45(12), 2481–2498. doi: 10.1017/s0033291715000574 25903153

[ref40] Turner, S., Harvey, C., Hayes, L., Castle, D., Galletly, C., Sweeney, S., … Spittal, M. J. (2019). Childhood adversity and clinical and psychosocial outcomes in psychosis. Epidemiology and Psychiatric Sciences, 29, e78. doi: 10.1017/s2045796019000684 31839014 PMC8061294

[ref41] van Nierop, M., Bak, M., de Graaf, R., Ten Have, M., van Dorsselaer, S., & van Winkel, R. (2016). The functional and clinical relevance of childhood trauma-related admixture of affective, anxious and psychosis symptoms. Acta Psychiatrica Scandinavica, 133(2), 91–101. doi: 10.1111/acps.12437 25961128

[ref42] van Nierop, M., Lataster, T., Smeets, F., Gunther, N., van Zelst, C., de Graaf, R., … van Winkel, R. (2014). Psychopathological mechanisms linking childhood traumatic experiences to risk of psychotic symptoms: Analysis of a large, representative population-based sample. Schizophrenia Bulletin, 40(Suppl 2), S123–S130. doi: 10.1093/schbul/sbt150 24562491 PMC3934395

[ref43] van Nierop, M., Viechtbauer, W., Gunther, N., van Zelst, C., de Graaf, R., ten Have, M., … van Winkel, R. (2015). Childhood trauma is associated with a specific admixture of affective, anxiety, and psychosis symptoms cutting across traditional diagnostic boundaries. Psychological Medicine, 45(6), 1277–1288. doi: 10.1017/S0033291714002372 25273550

[ref44] Varese, F., Barkus, E., & Bentall, R. P. (2012). Dissociation mediates the relationship between childhood trauma and hallucination-proneness. Psychological Medicine, 42(5), 1025–1036. doi: 10.1017/s0033291711001826 21896238

[ref45] Varese, F., Smeets, F., Drukker, M., Lieverse, R., Lataster, T., Viechtbauer, W., … Bentall, R. P. (2012). Childhood adversities increase the risk of psychosis: A meta-analysis of patient-control, prospective- and cross-sectional cohort studies. Schizophrenia Bulletin, 38(4), 661–671. doi: 10.1093/schbul/sbs050 22461484 PMC3406538

[ref46] Vila-Badia, R., Butjosa, A., Del Cacho, N., Serra-Arumí, C., Esteban-Sanjusto, M., Ochoa, S., & Usall, J. (2021). Types, prevalence and gender differences of childhood trauma in first-episode psychosis. What is the evidence that childhood trauma is related to symptoms and functional outcomes in first-episode psychosis? A systematic review. Schizophrenia Research, 228, 159–179. doi: 10.1016/j.schres.2020.11.047 33434728

[ref47] Wallwork, R. S., Fortgang, R., Hashimoto, R., Weinberger, D. R., & Dickinson, D. (2012). Searching for a consensus five-factor model of the Positive and Negative Syndrome Scale for schizophrenia. Schizophrenia Research, 137(1–3), 246–250. doi: 10.1016/j.schres.2012.01.031 22356801 PMC3351536

[ref48] WHO Collaborating Centre for Drug Statistics Methodology (2013). Guidelines for ATC classification and DDD assignment 2014. Oslo: World Health Organization.

